# Supplementary material for: Orthogonal proteogenomic analysis identifies the druggable PA2G4-MYC axis in 3q26 AML
Source: Nat Commun. 2024 Jun 4;15:4739. doi: 10.1038/s41467-024-48953-3 (PMC11150407; doi:10.1038/s41467-024-48953-3)
Supplement: Supplementary file 1 — Supplementary Information [file 41467_2024_48953_MOESM1_ESM.pdf]

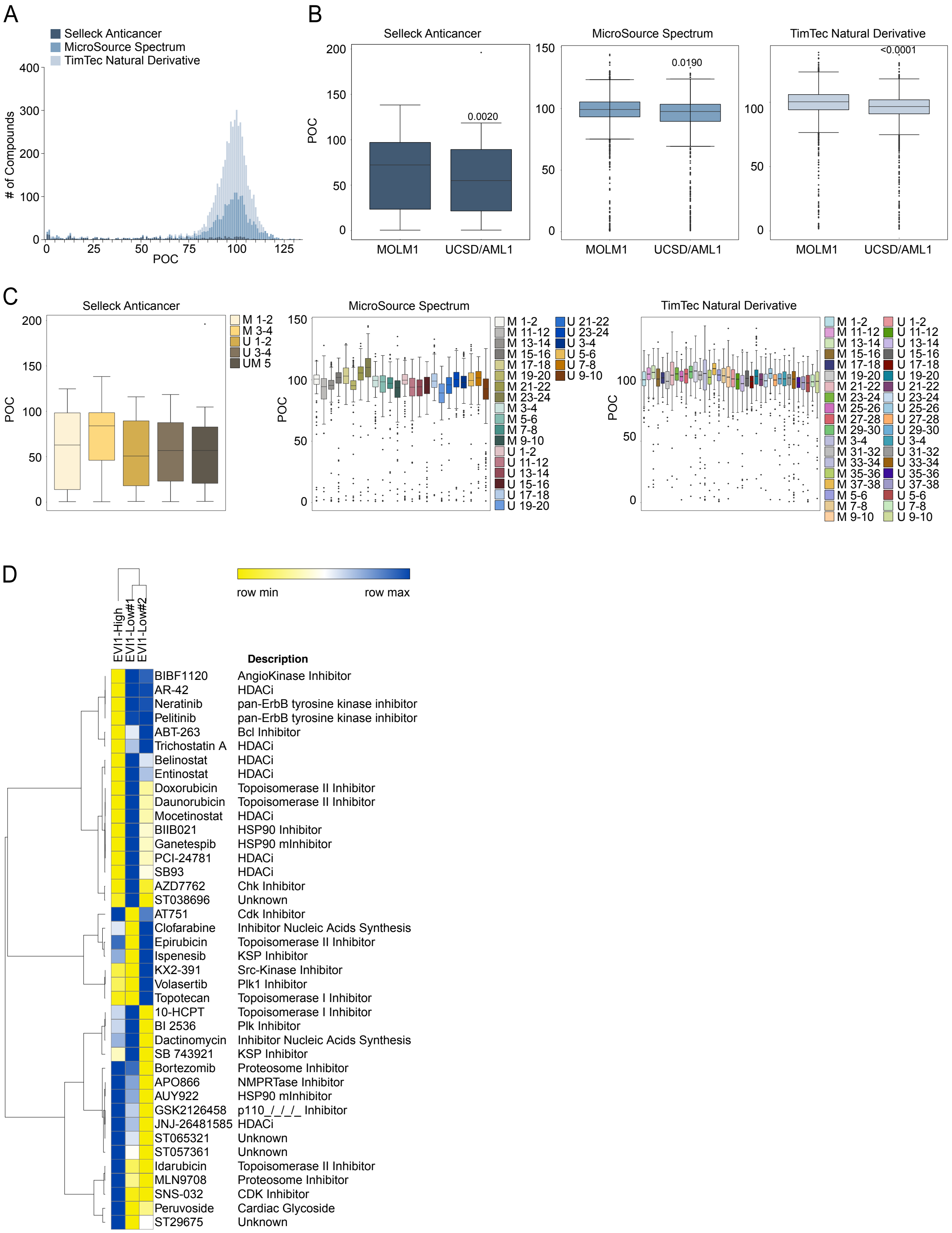
A
Selleck Anticancer
MicroSource Spectrum
TimTec Natural Derivative
# of Compounds
POC
B
Selleck Anticancer
MicroSource Spectrum
TimTec Natural Derivative
POC
0.0020
0.0190
<0.0001
MOLM1
UCSD/AML1
C
Selleck Anticancer
MicroSource Spectrum
TimTec Natural Derivative
POC
M 1-2
M 3-4
U 1-2
U 3-4
UM 5
D
row min
row max
EVI1-High
EVI1-Low#1
EVI1-Low#2
Description
BIBF1120 AngioKinase Inhibitor
AR-42 HDACi
Neratinib pan-ErbB tyrosine kinase inhibitor
Pelitinib pan-ErbB tyrosine kinase inhibitor
ABT-263 Bcl Inhibitor
Trichostatin A HDACi
Belinostat HDACi
Entinostat HDACi
Doxorubicin Topoisomerase II Inhibitor
Daunorubicin Topoisomerase II Inhibitor
Mocetinostat HDACi
BIIB021 HSP90 Inhibitor
Ganetespib HSP90 mInhibitor
PCI-24781 HDACi
SB93 HDACi
AZD7762 Chk Inhibitor
ST038696 Unknown
AT751 Cdk Inhibitor
Clofarabine Inhibitor Nucleic Acids Synthesis
Epirubicin Topoisomerase II Inhibitor
Ispenesib KSP Inhibitor
KX2-391 Src-Kinase Inhibitor
Volasertib Plk1 Inhibitor
Topotecan Topoisomerase I Inhibitor
10-HCPT Topoisomerase I Inhibitor
BI 2536 Plk Inhibitor
Dactinomycin Inhibitor Nucleic Acids Synthesis
SB 743921 KSP Inhibitor
Bortezomib Proteosome Inhibitor
APO866 NMPRTase Inhibitor
AUY922 HSP90 mInhibitor
GSK2126458 p110_/_/_/_ Inhibitor
JNJ-26481585 HDACi
ST065321 Unknown
ST057361 Unknown
Idarubicin Topoisomerase II Inhibitor
MLN9708 Proteosome Inhibitor
SNS-032 CDK Inhibitor
Peruvoside Cardiac Glycoside
ST29675 Unknown

Supplementary Figure 1 (related to Figure 1 and Supplementary Data 1). Identification of HDAC inhibitors in 3q26 AML

(A) Histogram showing the distribution of percentage over control (POC) values for the Selleck anticancer (dark blue), MicroSource Spectrum (dark turquoise), and TimTec Natural Derivative (cyan) compounds. The effect on viability was normalized to vehicle control (DMSO) and plotted on the x axis. The y axis indicates the number of small molecules with values falling in the POC intervals.

(B) POC effect in MOLM1 and UCSD/AML1 3q26 AML cell lines. The line in the box-and-whisker plot represents the median centered ratio. The box's upper edge (hinge) indicates the 75th percentile of the data, and the lower hinge represents the 25th percentile. The whiskers represent the minimum and maximum data values. All small molecule libraries were tested in duplicates ± standard deviation (SD). Statistical significance was determined by a two-sided non-parametric t-test (Mann-Whitney).

(C) Box-and-whisker plot showing POC's effect in MOLM1 (M) and UCSD/AML1 (U) AML cell lines by row plates. All small-molecules libraries were tested in n=2 biological replicates.

(D) Heatmap showing the effect AUC of 40 hit compounds (listed in **Supplementary Data** 1). Columns represent AML cell lines, and rows represent small molecules. The color code indicates the range of relative effects for a given molecule. Unsupervised clustering was computed by calculating Pearson correlation coefficients.

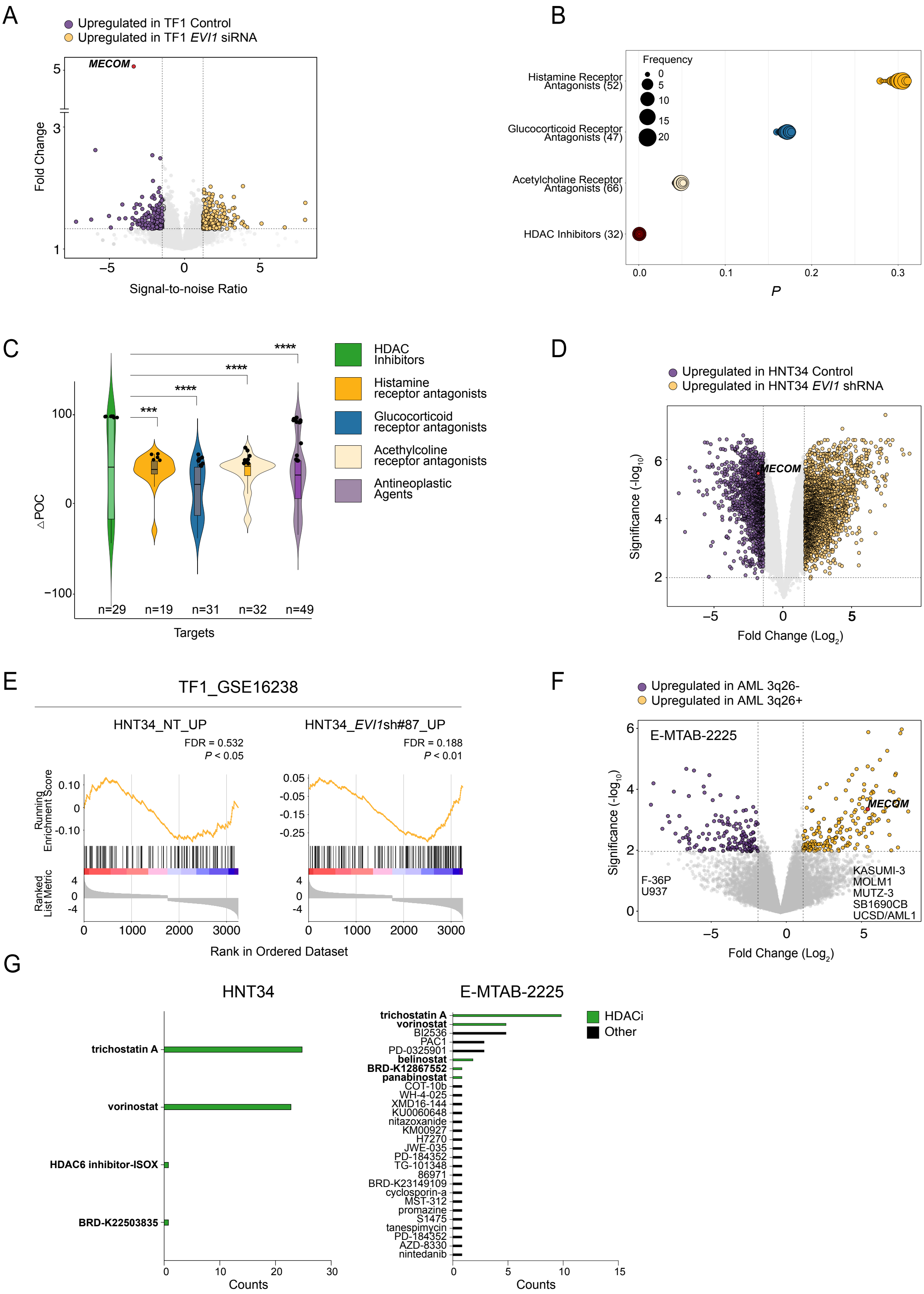
A
Upregulated in TF1 Control
Upregulated in TF1 EVI1 siRNA
MECOM
Fold Change
Signal-to-noise Ratio
B
Frequency
0
5
10
15
20
Histamine Receptor Antagonists (52)
Glucocorticoid Receptor Antagonists (47)
Acetylcholine Receptor Antagonists (66)
HDAC Inhibitors (32)
P
C
HDAC Inhibitors
Histamine receptor antagonists
Glucocorticoid receptor antagonists
Acethylcoline receptor antagonists
Antineoplastic Agents
△POC
n=29
n=19
n=31
n=32
n=49
Targets
D
Upregulated in HNT34 Control
Upregulated in HNT34 EVI1 shRNA
MECOM
Significance (-log10)
Fold Change (Log2)
E
TF1_GSE16238
HNT34_NT_UP
FDR = 0.532
P < 0.05
HNT34_EVI1sh#87_UP
FDR = 0.188
P < 0.01
Running Enrichment Score
Ranked List Metric
Rank in Ordered Dataset
F
Upregulated in AML 3q26-
Upregulated in AML 3q26+
E-MTAB-2225
MECOM
F-36P
U937
KASUMI-3
MOLM1
MUTZ-3
SB1690CB
UCSD/AML1
Significance (-log10)
Fold Change (Log2)
G
HNT34
trichostatin A
vorinostat
HDAC6 inhibitor-ISOX
BRD-K22503835
Counts
E-MTAB-2225
trichostatin A
vorinostat
BI2536
PAC1
PD-0325901
belinostat
BRD-K12867552
panabinostat
COT-10b
WH-4-025
XMD16-144
KU0060648
nitazoxanide
KM00927
H7270
JWE-035
PD-184352
TG-101348
86971
BRD-K23149109
cyclosporin-a
MST-312
promazine
S1475
tanespimycin
PD-184352
AZD-8330
nintedanib
Counts
HDACi
Other

Supplementary Figure 2 (related to Figure 1 and Supplementary Data 1). Gene-expression signature approaches identify inducer of an EVI1 "*Off*" state.

(A) Volcano plot derived from TF1 cells gene expression data (Affymetrix microarray profiling from E-GEOD 16238[1]) after treatment with an *EVI1*-directed siRNA (*EVI1* siRNA n=3) or non-targeting control (Control n=3). DEGs are depicted in dark yellow if upregulated in *EVI1* siRNA (SNR ≥ 1.5, *Adj.P* ≤ 0.01) or in violet if upregulated in Control (SNR ≤ - 1.5 or, *Adj.P* ≤ 0.01).

(B) Average *P* value (x axis) for molecules of interest (MOIs; y axis) computed by Wilcoxon rank-sum test by comparing 10,000 random drug sets against MOIs and performing 100 iterations.

(C) Violin plots comparing ΔPOC activity between HDACis and the indicated drug classes. "n" indicates the number of small molecules within each class (HDACi n=29, histamine receptor antagonist n=19, glucocorticoid receptor antagonists n=31, acethylcoline receptor antagonists n=32, antineoplastic agents n=49). Statistical significance among groups was determined by one-way ANOVA (C) using Tukey's correction for multiple comparison testing.

(D) Volcano plot derived from RNASeq gene expression data of HNT34 cells transduced with a non-targeting shRNA (Control, n=3) or after *EVI1*-directed shRNAs (sh#16, n=3 and sh#87, n=3) three days after selection (see also **Supplementary Figure 3A**). DEGs are depicted in violet if upregulated in Control ($\log_2$ fold change ≤ - 2, *Adj.P* ≤ 0.05) or in dark yellow if upregulated in *EVI1* shRNA ($\log_2$ fold change ≥ 2, *Adj.P* ≤ 0.05).

(E) GSEA analysis of Affymetrix data from **Supplementary Figure 1E** using the oncogenic gene sets from HNT34 transduced with shRNAs targeting *EVI1*. Enrichment plot of the *EVI1* overlapping genes is shown. GSEA enrichment score significance was based on a weighted Kolmogorov Smirnov (WKS) test corrected for multiple hypotheses testing: Benjamini & Hochberg (BH or FDR) [2]. NES, normalized enrichment score; FDR, false discovery rate.

(F) Volcano plot of RNASeq expression in $EVI1^{High}$ (n=5) or $EVI1^{Low}$ (n=2) AML cell lines segregated based on the 3q26 status derived from the E-MTAB-2225 dataset [3]. From DEGs upregulated genes in $EVI1^{High}$ (n=8247) and upregulated in $EVI1^{Low}$ (n=7699) are depicted those with $\log_2$ fold change ≤ - 2 (n=118, violet) and with a $\log_2$ fold change ≥ 2 (n=144, dark yellow).

(G) Bar plot displaying top drugs inducing an *EVI1* “*Off*” status identified by the L1000CDS^2 query of HNT34 (from **Supplementary Figure 2D**, left) and E-MTAB-2225 (from **Supplementary Figure 2F**, right) datasets. HDAC inhibitors are highlighted in green.

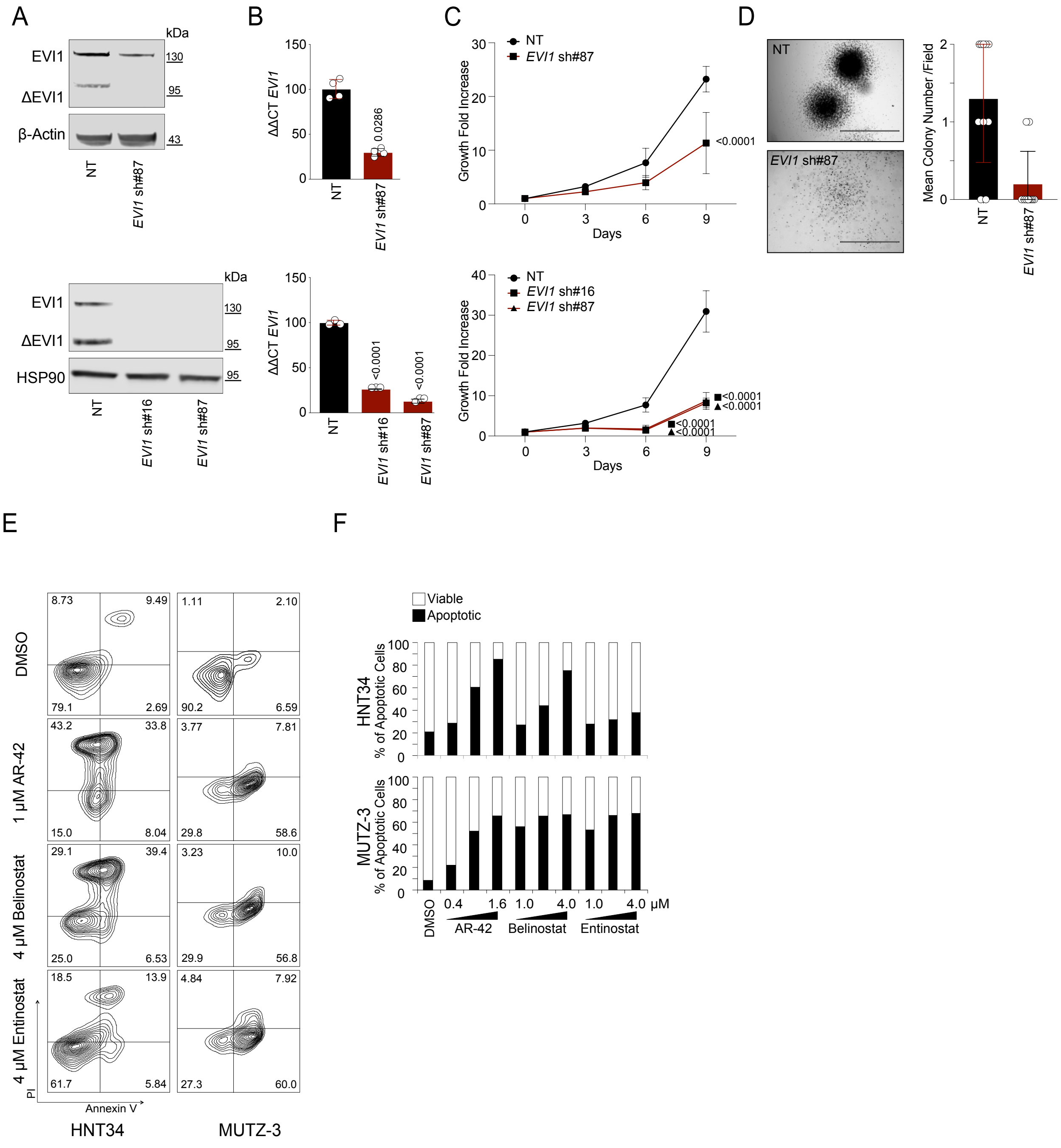
A
B
C
D
E
F
EVI1
ΔEVI1
β-Actin
HSP90
kDa
130
95
43
NT
EVI1 sh#87
EVI1 sh#16
ΔΔCT EVI1
0.0286
<0.0001
Growth Fold Increase
Days
<0.0001
Mean Colony Number /Field
Viable
Apoptotic
HNT34
MUTZ-3
% of Apoptotic Cells
DMSO
1 μM AR-42
4 μM Belinostat
4 μM Entinostat
PI
Annexin V
AR-42
Belinostat
Entinostat
μM

Supplementary Figure 3 (related to Figure 2). Effect of *EVI1* loss in 3q26 AML

(A) Western blot analysis showing the expression of EVI1 and ΔEVI1 in UCSD/AML1 (top panel) or HNT34 (bottom panel) cells six days after shRNA transduction (n=2 biological replicates).

(B) Percentage of *EVI1* mRNA relative to the control gene *RPL13A* (ΔΔCT) in UCSD/AML1 (top panel) or HNT34 (bottom panel) cells six days after shRNA transduction.

(C) Effect of *EVI1* loss in UCSD/AML1 (top panel) or HNT34 (bottom panel) cells at three, six, or nine days after shRNA selection.

NT = non-targeting, sh#16 and #87 = shRNAs directed against *EVI1* (A, B, C).

(D) Effect of shRNA mediated *EVI1* loss on UCSD/AML1 ability to form colonies in methylcellulose (n=2 biological replicates). Scale bar: 1000 μm.

(E) Annexin V/propidium iodide (PI) staining after 72 hr of HDACis treatment in HNT34 and MUTZ-3. Events ≥ 20.000.

(F) Apoptotic fold increase expressed as a percentage of annexin V-positive cells relative to vehicle-control.

Statistical significance among groups was determined by a two-sided non-parametric t-test (Mann-Whitney) (B upper panel) or one-way (B lower panel), two-way (C) ANOVA using Tukey's correction for multiple comparison testing. Data are presented as mean ± SD in B (n=4 upper panel, n=3 lower panel), C (n=5), D (n=10 fields per condition). Source data are provided as a Source Data file.

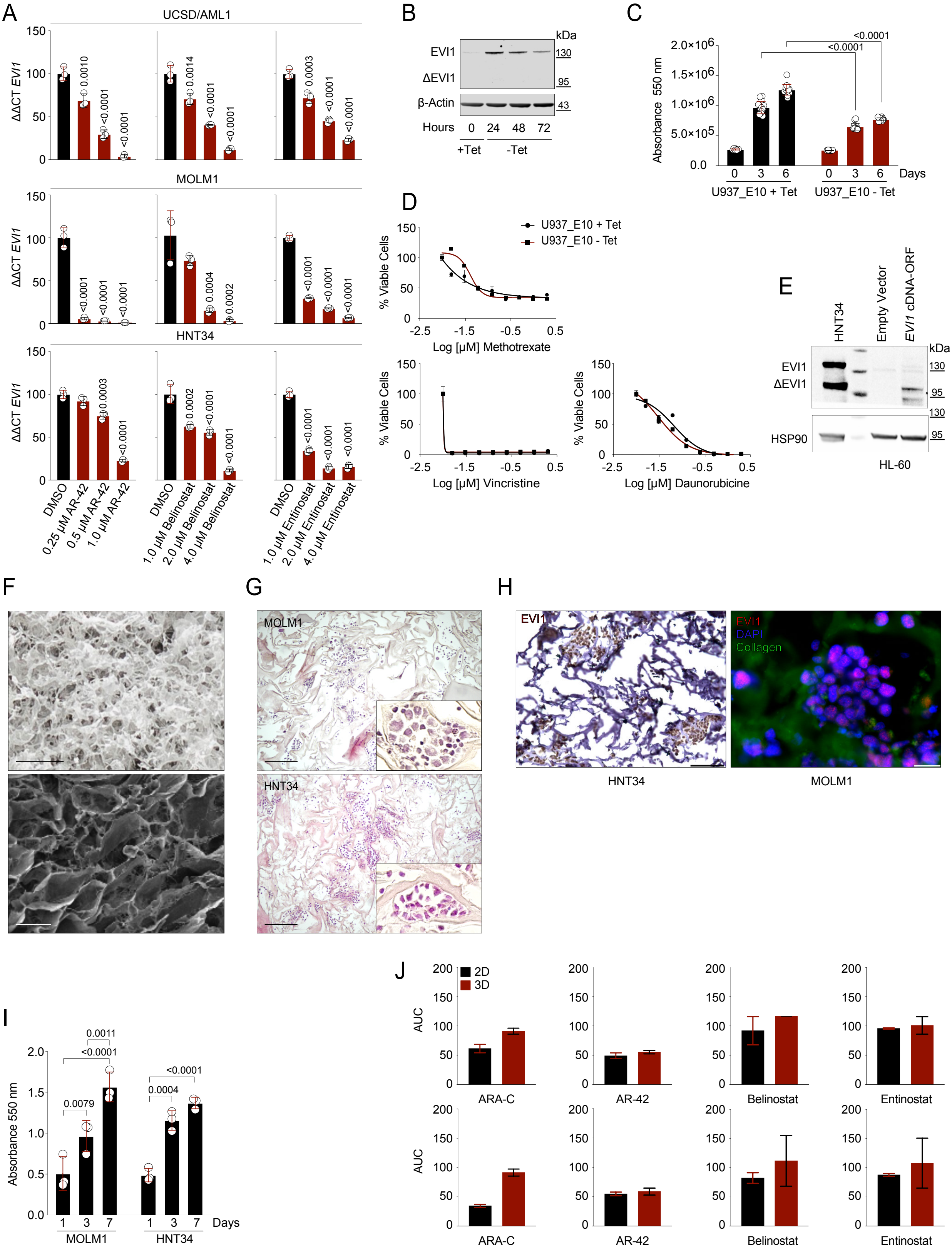

A
UCSD/AML1
MOLM1
HNT34
ΔΔCT EVI1
DMSO
0.25 µM AR-42
0.5 µM AR-42
1.0 µM AR-42
1.0 µM Belinostat
2.0 µM Belinostat
4.0 µM Belinostat
1.0 µM Entinostat
2.0 µM Entinostat
4.0 µM Entinostat
B
EVI1
ΔEVI1
β-Actin
kDa
Hours 0 24 48 72
+Tet -Tet
C
Absorbance 550 nm
Days
U937_E10 + Tet
U937_E10 - Tet
D
U937_E10 + Tet
U937_E10 - Tet
% Viable Cells
Log [µM] Methotrexate
Log [µM] Vincristine
Log [µM] Daunorubicine
E
HNT34
Empty Vector
EVI1 cDNA-ORF
EVI1
ΔEVI1
HSP90
HL-60
F
G
MOLM1
HNT34
H
EVI1
DAPI
Collagen
HNT34
MOLM1
I
Absorbance 550 nm
Days
MOLM1
HNT34
J
2D
3D
AUC
ARA-C
AR-42
Belinostat
Entinostat

Supplementary Figure 4 (related to Figure 3). HDACis modulate EVI1 in 3q26 leukemia models

(A) Percentage of *EVI1* mRNA relative to the control gene *RPL13A* (ΔΔCT) in $EVI1^{High}$ AML cell lines.

(B) EVI1 and ΔEVI1 expression in U937T_E10 cells in the presence or absence of tetracycline (n=3 biological replicates).

(C) Effect of EVI1 expression on cell proliferation in U937T_E10 in the presence ($EVI1^{Low}$) or absence of tetracycline ($EVI1^{High}$). Histograms show the ATP absorbance fold increase relative to vehicle control.

(D) Effect of methotrexate, vincristine, and daunorubicin on cell viability after 72 hr of treatment in U937T_E10 cultured with (black line, $EVI1^{Low}$) or without (red line, $EVI1^{High}$) tetracycline.

(E) Western blot analysis showing HL-60 AML cell line transduced with an empty vector or an ORF-*EVI1* cDNA plasmid (n=2 biological replicates).

(F) Top panel: representative images of collagen type I scaffolds (n=3 imaged scaffolds). Scale bar: 500 μm. Bottom panel: scanning electron microscope (SEM) scaffold micrograph showing the collagen fibers' architecture and the biological matrix's porosity. Scale bar: 100 μm.

(G) Hematoxylin-and-eosin–stained (H&E) histological sections of $EVI1^{High}$ 3q26 AML cell line grown in collagen type I scaffolds for 7 days (n=3 imaged scaffolds per cell line). Insets show niche-like structures. Scale bar: 100 μm.

(H) Expression of EVI1 in 3q26 AML cell lines in 3D models. Left: representative immunohistochemical staining (IHC) for EVI1 (brownish) in HNT34 cells and hematoxylin-and-eosin staining (H&E) for the collagen matrix (n=3 imaged scaffolds). Scale bar: 50 μm. Right: immunofluorescence images of MOLM1 grown for 7 days in 3D collagen type 1 scaffold incubated with anti-EVI1 (in red) (n=2 imaged scaffolds). Nuclei were stained with DAPI (blue) and the collagen matrix was detected by autofluorescence (green). Scale bar: 20 μm.

(I) Histograms showing proliferation fold increase based on MTT absorbance relative to day 0 in $EVI1^{High}$ MOLM1 and HNT34 cells grown in collagen type I scaffolds.

(J) AUC effect of ara-C, AR-42, belinostat, and entinostat in EVI1^{High} MOLM1 (top) and HNT34 (bottom) grown in standard 2D or 3D collagen type I scaffold cell culture. Cell viability was assessed after 72 hr using the same concentration in 2D or 3D cell cultures.

Statistical significance among groups was determined by one-way (A) or two-way (C, I) ANOVA using Tukey's correction for multiple comparison testing. Data are presented as mean ± SD in A (n=3), C (n=14), D (n=2), I (n=3), J (n=2). Source data are provided as a Source Data file.

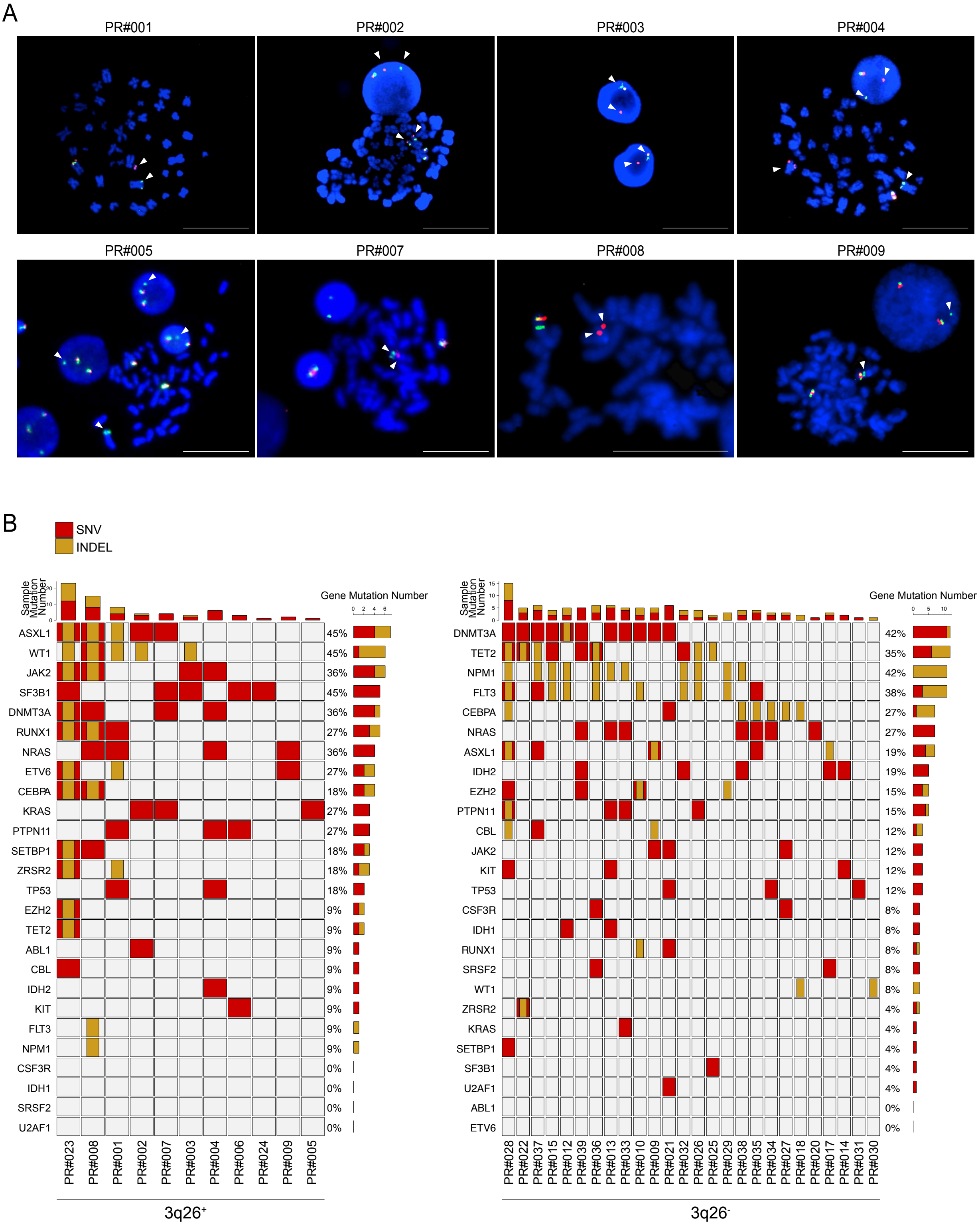

A
PR#001
PR#002
PR#003
PR#004
PR#005
PR#007
PR#008
PR#009
B
SNV
INDEL
Sample Mutation Number
Gene Mutation Number
ASXL1
WT1
JAK2
SF3B1
DNMT3A
RUNX1
NRAS
ETV6
CEBPA
KRAS
PTPN11
SETBP1
ZRSR2
TP53
EZH2
TET2
ABL1
CBL
IDH2
KIT
FLT3
NPM1
CSF3R
IDH1
SRSF2
U2AF1
PR#023
PR#008
PR#001
PR#002
PR#007
PR#003
PR#004
PR#006
PR#024
PR#009
PR#005
3q26+
DNMT3A
TET2
NPM1
FLT3
CEBPA
NRAS
ASXL1
IDH2
EZH2
PTPN11
CBL
JAK2
KIT
TP53
CSF3R
IDH1
RUNX1
SRSF2
WT1
ZRSR2
KRAS
SETBP1
SF3B1
U2AF1
ABL1
ETV6
3q26-

Supplementary Figure 5 (related to Figure 4 and Supplementary Data 2). Genomic, genetic, and clinical characteristics of 3q26 AML cases enrolled in this study

(A) Representative abnormal 3q26 pattern on fluorescence in situ hybridization (FISH) in PR#001-005 and PR#007-009. The break-apart hybridization pattern 1F1G1O (one fusion and two separated signals, one green and one orange) indicates the break and split of the *EVI1* locus. The abnormal pattern was observed in 100% of blasts analyzed for PR#001, 95% for PR#003 and PR#004, 90% for PR#009, 89% for PR#002, 80% for PR#008, 73% for PR#005, and 30% for PR#006 and PR#007, and 97% for PR#023, 80% for PR#024 (n=100 nuclei/patients were analyzed). FISH representative images were not available for PR#006, PR#023 and for PR#024. Scale bar: 100 µm.

(B) Oncoprint plot showing somatic mutations in recurrent mutated genes (n=30) in AML. Left, AML cases carrying 3q26 abnormalities (n=11, PR#001-009 and PR#023-024); right, other AML subtypes (n=28, PR#010-022 and PR#025-039). Single nucleotide variations (SNVs, in red) or insertions/deletions (INDELs, in yellow) of sequenced genes are listed on the left of the plots. Histograms show the event frequency of SNVs or INDELs detected in each sample (on the top) or for each gene in the cohort (right, percentage of mutation is also indicated). No mutations were detected for *BRAF, CALR*, *CSFR3R*, *HRAS*, and *MPL* genes in our cohort. NGS data were not available for PR#011 and PR#016.

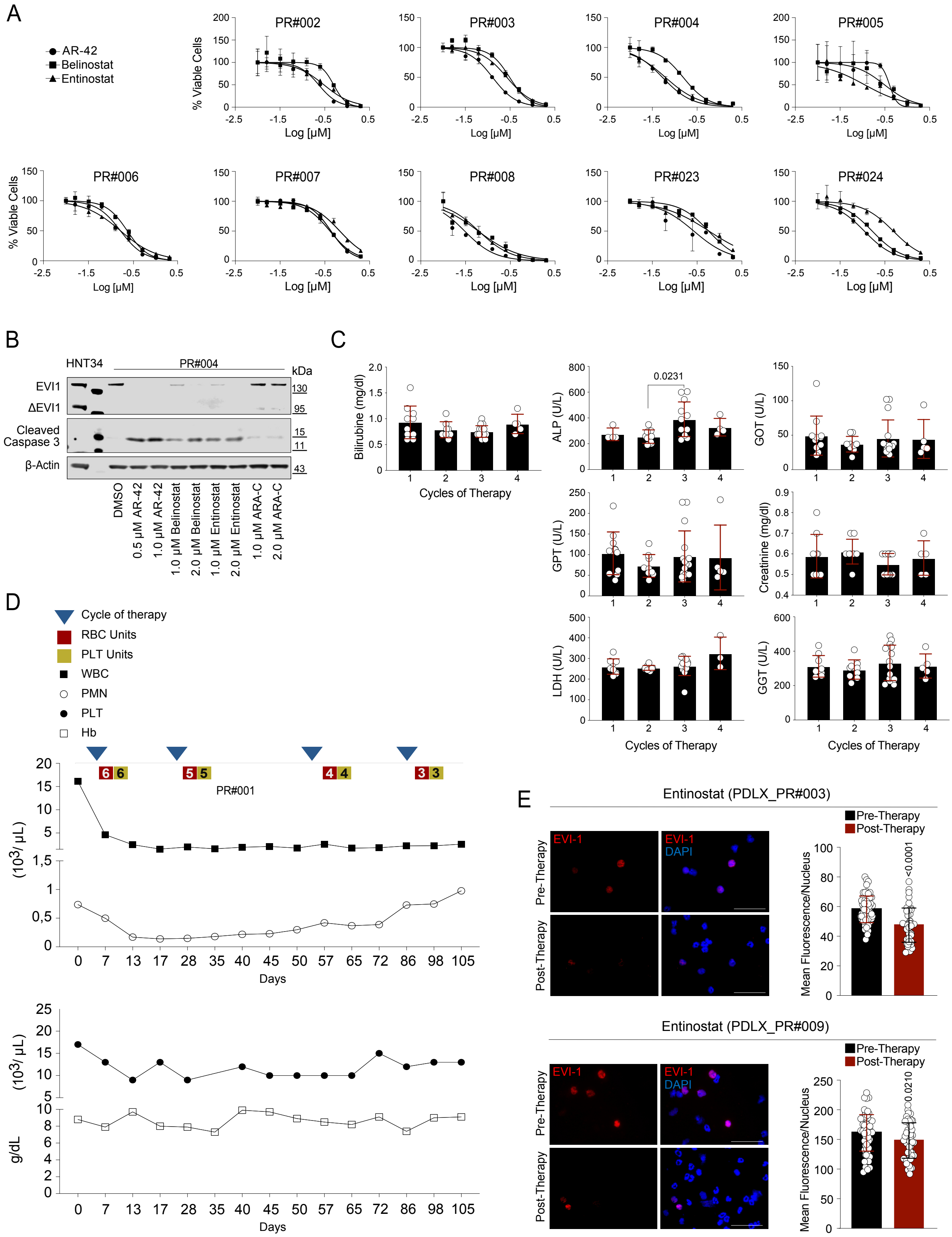

A
AR-42
Belinostat
Entinostat
% Viable Cells
Log [µM]
PR#002
PR#003
PR#004
PR#005
PR#006
PR#007
PR#008
PR#023
PR#024
B
HNT34
PR#004
kDa
EVI1
ΔEVI1
Cleaved Caspase 3
β-Actin
130
95
15
11
43
DMSO
0.5 µM AR-42
1.0 µM AR-42
1.0 µM Belinostat
2.0 µM Belinostat
1.0 µM Entinostat
2.0 µM Entinostat
1.0 µM ARA-C
2.0 µM ARA-C
C
Bilirubine (mg/dl)
ALP (U/L)
0.0231
GOT (U/L)
GPT (U/L)
Creatinine (mg/dl)
LDH (U/L)
GGT (U/L)
Cycles of Therapy
D
Cycle of therapy
RBC Units
PLT Units
WBC
PMN
PLT
Hb
PR#001
(10³/ µL)
g/dL
Days
E
Entinostat (PDLX_PR#003)
Entinostat (PDLX_PR#009)
Pre-Therapy
Post-Therapy
EVI-1
DAPI
Mean Fluorescence/Nucleus
<0.0001
0.0210

Supplementary Figure 6 (related to Figure 4 and Supplementary Data 2). Effect of HDACis on clinical features, and patient derived 3q26 AML cells and PDLX

(A) Effect of AR-42, belinostat, and entinostat on cell viability after 72 hr of treatment in available 3q26 AML cells, as assessed by an ATP-based luminescence viability assay. Error bars denote ± standard deviation (SD) of two replicates.

(B) Western blot analysis showing EVI1, ΔEVI1, and cleaved caspase 3 expression in PR#004 after 24 hr of treatment with the indicated concentrations of HDACis and ara-C. HNT34 cells were loaded as a positive control.

(C) Extra-hematologic toxicity of HDACi-based therapy in patients with 3q26 AML. Mean concentrations of aspartate aminotransferase (GOT), bilirubin, alanine aminotransferase (GPT), lactate dehydrogenase (LDH), gamma-glutamyl transferase (GGT), alkaline phosphatase (ALP), and creatinine in PR#002 during four cycles of azacitidine at 50 mg/m^2 and entinostat at 4 mg/m^2.

(D) Cell blood count of PR#001 receiving azacitidine 50 mg/m^2 every (day 1-10) and entinostat 4 mg/m^2 on days 3 and 10 every 28 days. The timeline at the top indicates cycles of therapy (blue triangle), transfusion units of red blood cells ([RBCs] red boxes), and platelets ([PLTs] yellow boxes) over time. Cell blood count of white blood cells (WBCs) and polymorphonuclear leukocytes (PMNs) in the middle. The bottom panel shows the blood concentration of PLT and hemoglobin (Hb).

(E) Effect of entinostat (10 mg/kg) on EVI1 nuclear localization (in red) following 6 hr of treatment in PDLX_PR#003 (n=3 biological replicates) and PDLX_PR#009 (n=3 biological replicates). The nuclei were stained with DAPI (blue). Scale bar: 100 µm. Histograms indicate the quantitative immunofluorescence analysis of EVI1 nuclear content before and after therapy. Scatter dot plots indicate the mean ± SD of fluorescence intensity for each nucleus analyzed and expressed as arbitrary units. Statistical significance was determined by a two-sided non-parametric t-test (Mann-Whitney).

Statistical significance among groups was determined by a two-sided non-parametric t-test (Mann-Whitney) (E) or one-way (C), ANOVA using Tukey's correction for multiple comparison testing. Data are presented as mean ± SD in C (n=41 GOT, n=41 GPT, n=37 GGT, n=29 ALP, n=38 bilirubin,

n=33 LDH, n=37 creatinine), E (n=103 PDLX_PR#003, n=128 PDLX_PR#009). Source data are provided as a Source Data file.

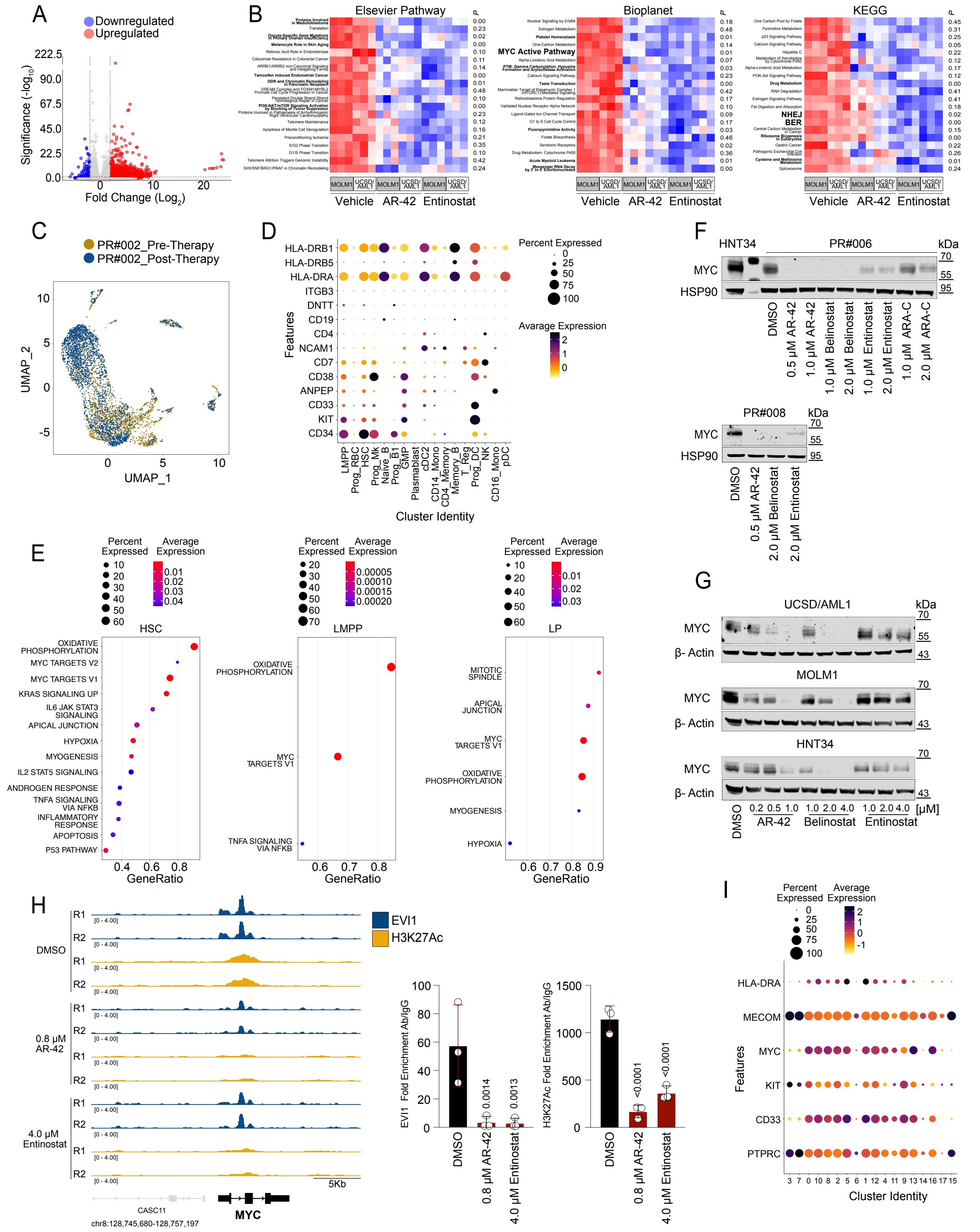

A
Downregulated
Upregulated
Significance (-log10)
Fold Change (Log2)
B
Elsevier Pathway
Bioplanet
KEGG
MYC Active Pathway
NHEJ
BER
Vehicle
AR-42
Entinostat
C
PR#002_Pre-Therapy
PR#002_Post-Therapy
UMAP_1
UMAP_2
D
Percent Expressed
Avarage Expression
Features
Cluster Identity
HLA-DRB1
HLA-DRB5
HLA-DRA
ITGB3
DNTT
CD19
CD4
NCAM1
CD7
CD38
ANPEP
CD33
KIT
CD34
F
HNT34
PR#006
kDa
MYC
HSP90
DMSO
0.5 µM AR-42
1.0 µM AR-42
1.0 µM Belinostat
2.0 µM Belinostat
1.0 µM Entinostat
2.0 µM Entinostat
1.0 µM ARA-C
2.0 µM ARA-C
PR#008
2.0 µM Belinostat
2.0 µM Entinostat
E
Percent Expressed
Average Expression
HSC
LMPP
LP
GeneRatio
OXIDATIVE PHOSPHORYLATION
MYC TARGETS V2
MYC TARGETS V1
KRAS SIGNALING UP
IL6 JAK STAT3 SIGNALING
APICAL JUNCTION
HYPOXIA
MYOGENESIS
IL2 STAT5 SIGNALING
ANDROGEN RESPONSE
TNFA SIGNALING VIA NFKB
INFLAMMATORY RESPONSE
APOPTOSIS
P53 PATHWAY
MITOTIC SPINDLE
G
UCSD/AML1
MOLM1
HNT34
MYC
β- Actin
DMSO
AR-42
Belinostat
Entinostat
[µM]
H
DMSO
0.8 µM AR-42
4.0 µM Entinostat
EVI1
H3K27Ac
5Kb
CASC11
MYC
chr8:128,745,680-128,757,197
EVI1 Fold Enrichment Ab/IgG
H3K27Ac Fold Enrichment Ab/IgG
0.0014
0.0013
<0.0001
<0.0001
I
Percent Expressed
Average Expression
Features
Cluster Identity
HLA-DRA
MECOM
MYC
KIT
CD33
PTPRC

Supplementary Figure 7 (related to Figure 5 and Supplementary Data 3). EVI1 modulates MYC in 3q26 AML

(A) Volcano plot of RNASeq expression following 16 hr of 0.5 µM AR-42, 2 µM entinostat in UCSD/AML1 or 0.8 µM AR-42, and 4 µM entinostat in MOLM1. DEG are depicted in blue if repressed (log_2 fold change ≤ - 2, *Adj.P* ≤ 0.05) or in red if upregulated (log_2 fold change ≥ + 2, *Adj.P* ≤ 0.05).

(B) Heatmap showing ssGSEA enrichment of Elsevier, KEGG and BioPlanet of gene signature in MOLM1 and UCSD/AML1 HDACi-treated cells. Hot or cold colors indicate correlation or anticorrelation of the top enriched gene sets from each functional group (in bold, *P* ≤ 0.05) among cell lines and treatments (bottom).

(C) UMAP plot of PR#002 scRNASeq data, colored according to therapy status.

(D) Dot plot illustrating gene expression (log of averaged counts) across the 17 cell clusters in the PR#002 scRNASeq data set. Genes defining the leukemia immunophenotype at diagnosis are included. The size of the dots represents the proportion of gene-expressing cells, and the color intensity represents the average level of gene expression.

(E) Dot plots of GSEA results illustrating Molecular Signatures Database (MsigDB) biological processes associated with EVI1 downregulation in HSC, LMPP, and LP in PR#002. Gene count refers to the number of genes associated with each (MsigDB) biological process. On the basis of co-expressed genes, the figures show the significant top positively and negatively enriched (MsigDB) terms (see **Supplementary Data 3**). Gene ratio (x axis) is the percentage of genes significantly correlated with EVI1 downregulation from the total number of genes associated with that process. Terms are ranked in the figure by decreasing gene ratio. Dot size is proportional to the number of matched genes within each pathway, while the color indicates the *Adj.P* for each pathway. GSEA enrichment score significance was based on a weighted Kolmogorov Smirnov (WKS) test corrected for multiple hypotheses testing: Benjamini & Hochberg (BH or FDR) [2].

(F) MYC expression following 24 hr of HDACi treatment in PR#006 and PR#008 bone marrow cells with the indicated concentrations of HDACis and ara-C.

(G) MYC expression following 24 hr of HDACi treatment in the 3q26 AML cell lines (n=2 biological replicates).

(H) On the left tracks showing EVI1 binding and H3K27ac enrichment across the *MYC* locus in HNT34 cells treated with DMSO, AR-42 and entinostat. The bottom bar represents the genes (hg19), and the y-axis represents normalized read density scaled to 1 million. On the right real-time PCR after ChIP performed in HNT34 cells treated with DMSO, AR-42 and entinostat at the indicated doses using an anti-EVI1 and H3K27Ac antibody and primers targeting the *MYC* promoter. Results are expressed as fold enrichment of *EVI1* and *H3K27Ac* compared to a non-specific IgG antibody. Statistical significance) was determined by ordinary one-way ANOVA using Tukey's correction for multiple comparison testing. Data are presented as mean ± SD (n=3).

(I) Dot plot illustrating gene expression (log of averaged counts) across the 17 cell clusters in the PDLX_PR#003 scRNASeq data set. Genes defining the leukemia immunophenotype at diagnosis are included. The size of the dots represents the proportion of gene-expressing cells, and the color intensity represents the average level of gene expression. Source data are provided as a Source Data file.

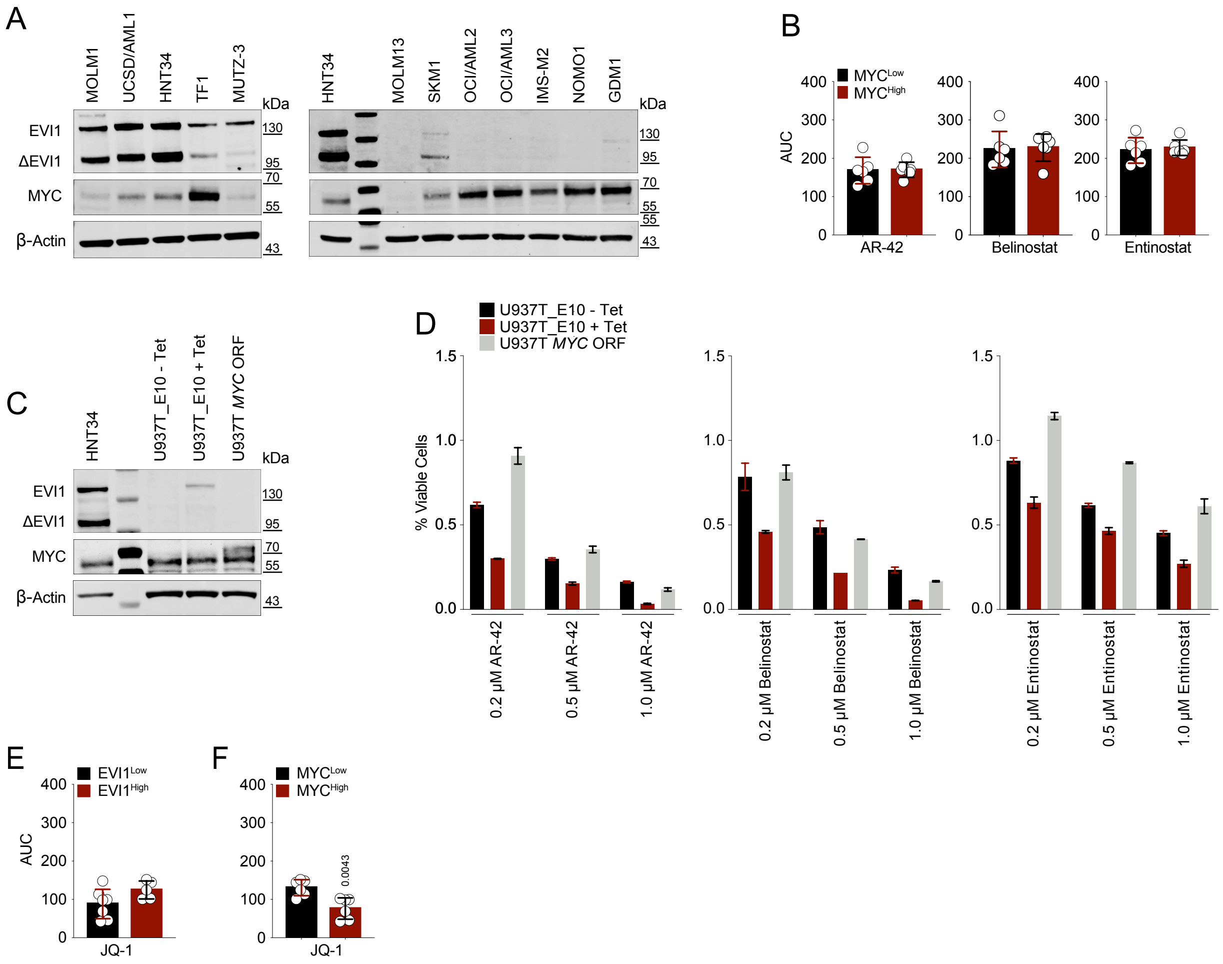

A
MOLM1
UCSD/AML1
HNT34
TF1
MUTZ-3
kDa
EVI1
ΔEVI1
MYC
β-Actin
130
95
70
55
43
HNT34
MOLM13
SKM1
OCI/AML2
OCI/AML3
IMS-M2
NOMO1
GDM1
B
MYCLow
MYCHigh
AUC
AR-42
Belinostat
Entinostat
C
HNT34
U937T_E10 - Tet
U937T_E10 + Tet
U937T MYC ORF
D
U937T_E10 - Tet
U937T_E10 + Tet
U937T MYC ORF
% Viable Cells
0.2 µM AR-42
0.5 µM AR-42
1.0 µM AR-42
0.2 µM Belinostat
0.5 µM Belinostat
1.0 µM Belinostat
0.2 µM Entinostat
0.5 µM Entinostat
1.0 µM Entinostat
E
EVI1Low
EVI1High
JQ-1
F
MYCLow
MYCHigh
0.0043
JQ-1

Supplementary Figure 8 (related to Figure 5 and Supplementary Data 3). MYC overexpression does not sensitize cells to HDAC inhibition in 3q26 AML models.

(A) EVI1, ∆EVI1, MYC and MYCN expression level in a panel of $EVI1^{High}$ (left) and $EVI1^{Low}$ (right) AML cell lines. β-Actin was used as loading control (n=3 biological replicates).

(B) Effect of AR-42, belinostat and entinostat on MYC^{High} (OCI/AML2, OCI/AML3, IMS-M2, GDM1, NOMO1 and TF1) and MYC^{Low} (SKM1, MOLM13, MOLM1, UCSD/AML1, HNT34 and MUTZ-3) AML cell lines. The AUC model of the log-transformed dose-response data is depicted.

(C) EVI1, ∆EVI1 and MYC expression level HNT34, U937T (clone E10) +/- tetracycline and *MYC* overexpressing U937T cells. β -Actin was used as loading control (n=3 biological replicates).

(D) Effects of AR-42, belinostat, and entinostat on viability in U937T +/- tetracycline and *MYC* overexpressing U937T cell lines following 72h of drug treatment at the indicated concentrations.

(E) Effect of JQ-1 on $EVI1^{High}$ (MOLM1, UCSD/AML1, HNT34, TF1 and MUTZ-3) and $EVI1^{Low}$ (NOMO1, MOLM13, OCI/AML2, OCI/AML3, GDM1, SKM1 and IMS-M2) AML cell lines calculated using the area under the curve (AUC) model of the log-transformed dose-response data.

(F) Effect of JQ-1 on MYC^{High} (OCI/AML2, OCI/AML3 IMS-M2, GDM1, NOMO1 and TF1) and MYC^{Low} (SKM1, MOLM13, MOLM1, UCSD/AML1, HNT34 and MUTZ-3) AML cell lines. The AUC model of the log-transformed dose-response data is depicted.

Statistical significance among groups was determined by a two-sided non-parametric t-test (Mann-Whitney) (B, E, F). Data are presented as mean ± SD in B (MYC^{High} =6, MYC^{Low}=6), D (n=2), E ($EVI1^{High}$=5, $EVI1^{Low}$=7), F (MYC^{High}=6, MYC^{Low}=6). Source data are provided as a Source Data file.

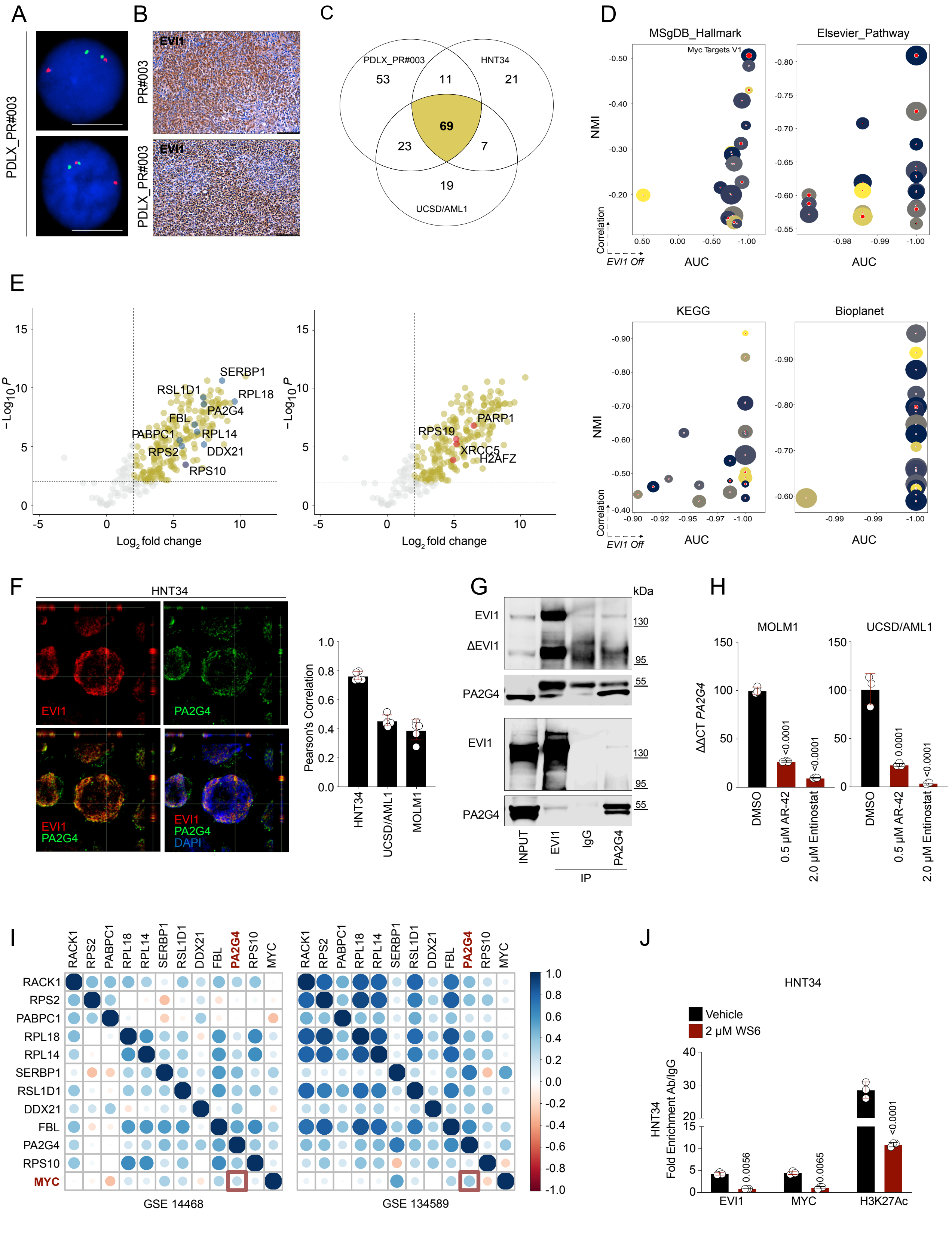

A
PDLX_PR#003
B
EVI1
PR#003
PDLX_PR#003
C
PDLX_PR#003
HNT34
UCSD/AML1
53
11
21
69
23
7
19
D
MSgDB_Hallmark
Myc Targets V1
Elsevier_Pathway
KEGG
Bioplanet
NMI
Correlation
EVI1 Off
AUC
E
Log2 fold change
−Log10 P
SERBP1
RSL1D1
RPL18
PA2G4
FBL
PABPC1
RPL14
RPS2
DDX21
RPS10
PARP1
RPS19
XRCC5
H2AFZ
F
HNT34
EVI1
PA2G4
DAPI
Pearson's Correlation
HNT34
UCSD/AML1
MOLM1
G
kDa
EVI1
ΔEVI1
PA2G4
INPUT
IgG
IP
H
MOLM1
UCSD/AML1
ΔΔCT PA2G4
DMSO
0.5 µM AR-42
2.0 µM Entinostat
<0.0001
I
RACK1
RPS2
PABPC1
RPL18
RPL14
SERBP1
RSL1D1
DDX21
FBL
PA2G4
RPS10
MYC
GSE 14468
GSE 134589
J
HNT34
Vehicle
2 µM WS6
HNT34 Fold Enrichment Ab/IgG
0.0056
0.0065
<0.0001
EVI1
MYC
H3K27Ac

Supplementary Figure 9 (related to Figure 6 and Supplementary Data 4). PA2G4 conveys Evi1/Myc signaling in 3q26 AML

(A) Abnormal 3q26 pattern on fluorescence in situ hybridization (FISH) in PR#003 cells grown subcutaneously in NSG mice. The break-apart hybridization pattern 1F1G1O (one fusion and two separated signals, one green and one orange) indicates the break and the split of the *MECOM* locus. Scale bar: 10 µm.

(B) Expression of EVI1 (brownish) in PR#003 and PDLX_PR#003. Formalin-fixed, paraffin-embedded (FFPE) tissue sections (top = bone marrow; bottom = subcutaneous xenotransplant) were stained with an anti-EVI1 antibody revealed by immunoperoxidase. Scale bar: 100 µm.

(C) Venn diagram showing the number of chromatin-associated proteins immunoprecipitated with an anti-EVI1 antibody by RIME HNT34 (n = 156), UCSD/AML1 (n = 108), and PR#003 (n = 118). Sixty-nine proteins were common to all samples.

(D) Bubble plot showing the intersection of ssGSEA enrichment of the Molecular Signatures Database (MSigDB), Elsevier, Kyoto Encyclopedia of Genes and Genomes (KEGG), and BioPlanet and RIME of 3q26 AML cells. The x axis represents the area under the curve (AUC) and the y axis the normalized mutual information ([NMI] y axis), calculated on the basis of ssGSEA. An NMI = 1 indicates an *EVI1* "*On*" status, and NMI = -1 indicates an *EVI1* "*Off*" status. The bubble size indicates the number of genes in each pathway per dataset, and the color scale indicates the level of statistical significance (t-*P*). The inner red dots show EVI1 interactors, identified by RIME, that are common in the genes contained in each pathway (**Supplementary Data 3**). Their size represents the number of EVI1-interacting proteins.

(E) Volcano plot displaying the enrichment of proteins in 3q26 AML samples (n=6) compared con control samples (n=6) identified by label free quantitation (LFQ). Statistical significance was performed with a two-sample t-test and with a permutation-based correction controlled with an FDR threshold of 0.05. Significant hits with a fold change > 4 and a $P < 0.01$ are depicted in yellow. In the left panel, blue-labeled proteins are MYC-pathway related proteins derived from the intersection of RIME and ssGSEA analysis as detailed in the manuscript. In the right panel, red-labeled proteins are EVI1 interactors previously identified using isotope labeling based MS.

(F) PA2G4 and EVI1 nuclear co-localization in 3q26 AML cell lines. Orthogonal views of x/y, x/z, and y/z planes from a representative z-stack image of HNT34 cells. EVI1 is labeled in red and PA2G4 in green; the cell nucleus was stained with DAPI (blue). Scale bars correspond to 20 µm. The dot plots on the right indicate the mean ± SD of the coefficient indexes of Pearson derived from the co-localization signal in HNT34, UCSD/AML1, and MOLM1 (n=5 nuclei/cell line).

(G) Co-immunoprecipitation of EVI1 and PA2G4 in HNT34 (top) and HEK-293T (bottom). HEK-293T were co-transfected with expression plasmid for *EVI1* and *PA2G4* (n=3 biological replicates).

(H) Percentage of *PA2G4* mRNA relative to the control gene *RPL13A* (ΔΔCT) in MOLM1 and UCSD/AML1 following HDACis treatment.

(I) Heat map showing the Spearman correlation coefficient of the pairwise comparison between genes in the indicated datasets, calculated on the basis of mas5 normalized data (GSE14468) or FPKM (GSE134589) of each gene.

(J) Chromatin immunoprecipitation assay on HNT34 treated with vehicle (DMSO) or 2 µM WS6 for 24 hr using an anti-EVI1, MYC, and H3K27Ac antibody. Real-time PCR primers target the E-Box DNA binding sites in the *PA2G4* gene promoter, 500 bp upstream of the transcription starting site region. Results are expressed as fold enrichment of *EVI1*, *MYC*, or *H3K27Ac* compared to a non-specific IgG antibody.

Statistical significance among groups was determined by one-way (H) or two-way (J) ANOVA using Tukey's correction for multiple comparison testing. Data are presented as mean ± SD in H (n=3) and in J (n=3). Source data are provided as a Source Data file.

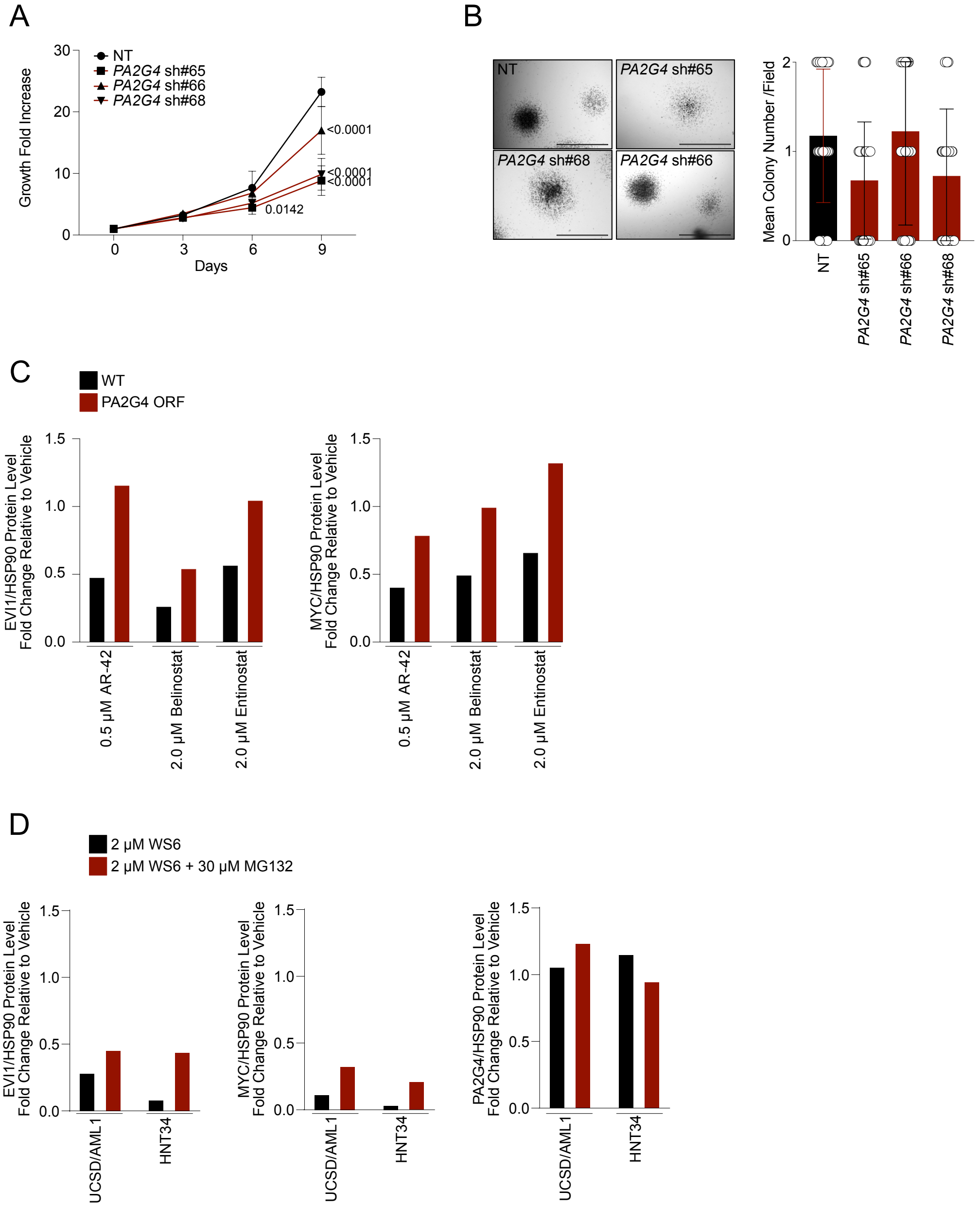

A
Growth Fold Increase
Days
NT
PA2G4 sh#65
PA2G4 sh#66
PA2G4 sh#68
<0.0001
<0.0001
<0.0001
0.0142
B
NT
PA2G4 sh#65
PA2G4 sh#68
PA2G4 sh#66
Mean Colony Number /Field
C
WT
PA2G4 ORF
EVI1/HSP90 Protein Level
Fold Change Relative to Vehicle
MYC/HSP90 Protein Level
Fold Change Relative to Vehicle
0.5 μM AR-42
2.0 μM Belinostat
2.0 μM Entinostat
D
2 μM WS6
2 μM WS6 + 30 μM MG132
EVI1/HSP90 Protein Level
MYC/HSP90 Protein Level
PA2G4/HSP90 Protein Level
Fold Change Relative to Vehicle
UCSD/AML1
HNT34

Supplementary Figure 10 (related to Figure 6 and Supplementary Data 4). Genetic modulation of *PA2G4* affects cell viability and rescues the HDACi-mediated effects

(A) Effect of *PA2G4* loss in UCSD/AML1 cells at three, six, or nine days after shRNA selection. NT = non-targeting, sh#65-68 = shRNAs directed against *PA2G4*.

(B) Effect of *PA2G4* loss in the ability of UCSD/AML1 to form colonies in methylcellulose. shRNAs directed against *PA2G4* inhibited colony formation compared to control (NT = non-targeting). Scale bar: 1000 µm. The histogram on the right shows the mean number of colonies per field 20 days after replating cells (n=2 biological replicates).

(C) Densitometric quantification of EVI1 and MYC proteins in wild-type or *PA2G4*-overexpressing HNT34 cells after 24 hr of treatment with HDACi at the indicated doses. The relative intensity of EVI1 and MYC was normalized for the levels of HSP90 and expressed as the fold change relative to wild type.

(D) Densitometric quantification of indicated proteins after WS6 treatment in the presence or absence of MG132. The relative intensity of EVI1, MYC, and PA2G4 was normalized for the levels of HSP90 and expressed as fold change relative to vehicle.

Statistical significance among groups was determined by two-way ANOVA (A) using Tukey's correction for multiple comparison testing respectively. Data are presented as mean ± SD A (n=5) in B (n=40 measurements for each condition). Source data are provided as a Source Data file.

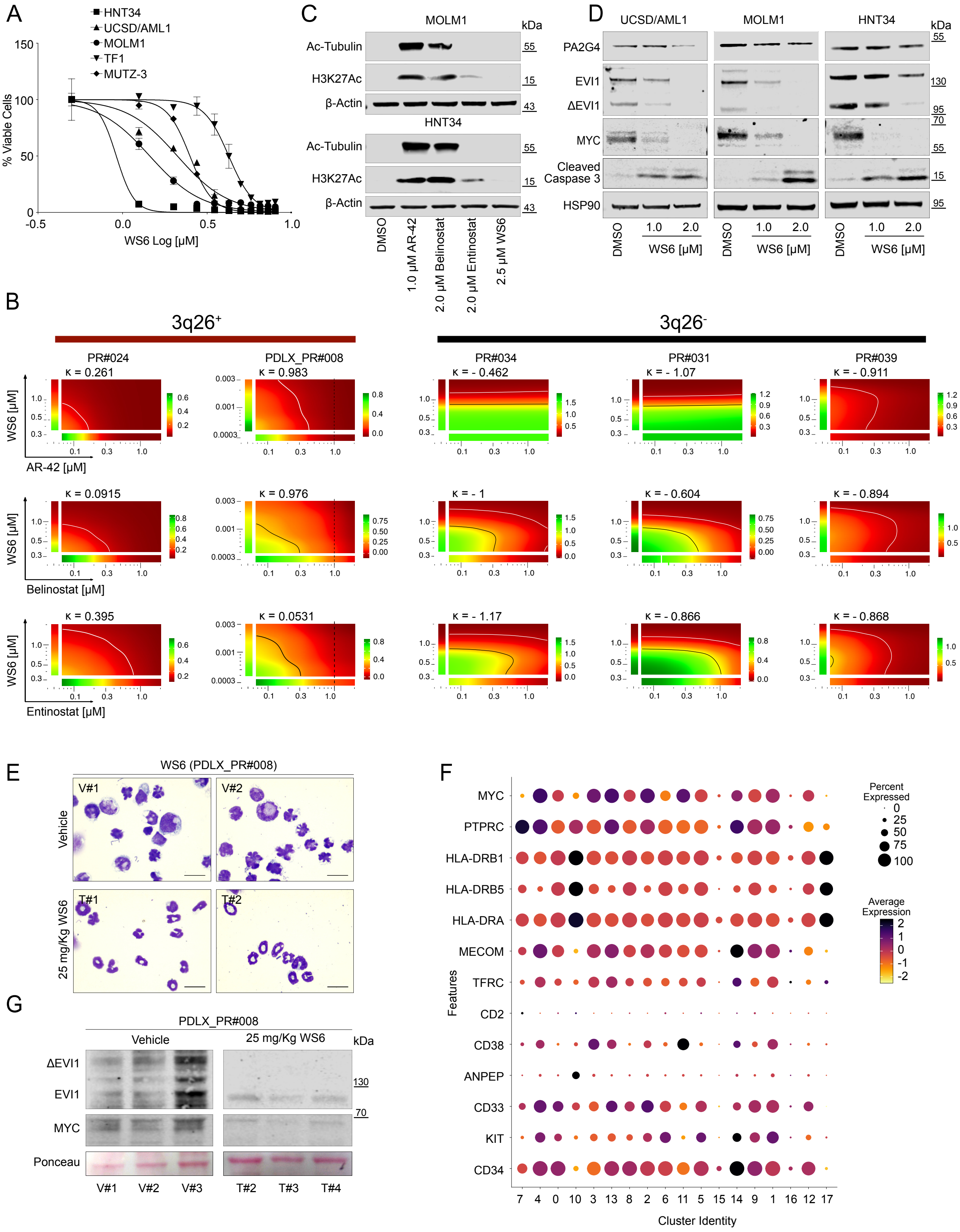
A
HNT34
UCSD/AML1
MOLM1
TF1
MUTZ-3
% Viable Cells
WS6 Log [μM]
C
MOLM1
HNT34
kDa
Ac-Tubulin
H3K27Ac
β-Actin
DMSO
1.0 μM AR-42
2.0 μM Belinostat
2.0 μM Entinostat
2.5 μM WS6
D
UCSD/AML1
MOLM1
HNT34
PA2G4
EVI1
ΔEVI1
MYC
Cleaved Caspase 3
HSP90
WS6 [μM]
B
3q26+
3q26-
PR#024
PDLX_PR#008
PR#034
PR#031
PR#039
WS6 [μM]
AR-42 [μM]
Belinostat [μM]
Entinostat [μM]
E
WS6 (PDLX_PR#008)
Vehicle
25 mg/Kg WS6
V#1
V#2
T#1
T#2
F
Percent Expressed
Average Expression
Features
MYC
PTPRC
HLA-DRB1
HLA-DRB5
HLA-DRA
MECOM
TFRC
CD2
CD38
ANPEP
CD33
KIT
CD34
Cluster Identity
G
PDLX_PR#008
Vehicle
25 mg/Kg WS6
ΔEVI1
EVI1
MYC
Ponceau
V#1
V#2
V#3
T#2
T#3
T#4

Supplementary Figure 11 (related to Figure 7). Chemical inhibition of PA2G4 abrogates the Evi1/Myc signaling axis

(A) Effects of WS6 treatment on viability in 3q26 AML cell lines following 72 hr drug treatment. Data are presented as mean ± SD (n=2).

(B) BRAID index analysis [4] for the combinations of WS6 with HDACi (AR-42, belinostat and entinostat) in n=2 EVI1^{High} (samples PR#024 and PDLX#PR008) and n=3 EVI1^{Low} (samples PR#034, PR#031 and PR#039) primary AML samples treated for 72h. A color scale bar represents the level of drug antagonism or synergism. K index is indicated. A positive K index indicates a synergistic effect.

(C) Acetylated tubulin (Ac-Tubulin) and H3K27Ac expression in 3q26 EVI1^{High} AML cell lines after 24 hr of treatment with the indicated concentrations of HDACis and WS6 (n=2 biological replicates).

(D) PA2G4, EVI1, ΔEVI1, MYC, and cleaved caspase 3 expression in 3q26 EVI1^{High} AML cell lines after 24 hr of treatment with DMSO or WS6 (n=2 biological replicates).

(E) Representative May Grünwald Giemsa staining of bone marrow blood cells collected from PDLX_PR#008 mice treated with DMSO (on the top) or 25 mg/Kg WS6 for 5 days/week for a total of 15 days (on the bottom). Representative n=2 mice per group are shown. Scale bar: 20 µm.

(F) Dot plot illustrating gene expression (log of averaged counts) in the n=17 cell clusters derived from scRNASeq of BM CD45^{+}-sorted AML cells (PDLX_PR#003). Genes defying the leukemia immunophenotype at diagnosis were included. The size of the dots represents the proportion of gene-expressing cells, and the color intensity of the dots represents the average level of gene expression.

(G) WS6 depletes EVI1 and MYC proteins *in vivo*. EVI1, ΔEVI1, and MYC protein expression in PDLX_PR#008 mice treated with vehicle (DMSO) or 25 mg/Kg WS6 5 days/week for a total of 15 days. Representative n=3 mice per group are shown. Ponceau staining was used as loading control. Source data are provided as a Source Data file.

Supplementary References

1. Vazquez I*, et al.* Down-regulation of EVI1 is associated with epigenetic alterations and good prognosis in patients with acute myeloid leukemia. *Haematologica* **96**, 1448-1456 (2011).

2. Subramanian A*, et al.* Gene set enrichment analysis: a knowledge-based approach for interpreting genome-wide expression profiles. *Proceedings of the National Academy of Sciences* **102**, 15545-15550 (2005).

3. Gröschel S*, et al.* A single oncogenic enhancer rearrangement causes concomitant EVI1 and GATA2 deregulation in leukemia. *Cell* **157**, 369-381 (2014).

4. Twarog NR, Stewart E, Hammill CV, Shelat AA. BRAID: A Unifying Paradigm for the Analysis of Combined Drug Action. *Sci Rep* **6**, 25523 (2016).
