# Supplementary material for: Orthogonal proteogenomic analysis identifies the druggable PA2G4-MYC axis in 3q26 AML
Source: Nat Commun. 2024 Jun 4;15:4739. doi: 10.1038/s41467-024-48953-3 (PMC11150407; doi:10.1038/s41467-024-48953-3)
Supplement: Supplementary file 3 — Description of Additional Supplementary Files [file 41467_2024_48953_MOESM3_ESM.pdf]

Description of additional Supplementary Files:

Supplementary Data 1:
Phenotypic and *in silico* small molecule screenings (Table S1.1-S1.6)

Supplementary Data 2:
Patient information and clinical history (Table S2.1)

Supplementary Data 3:
Gene expression analysis (RNASeq, scRNASeq, Table S3.1-S3.15)

Supplementary Data 4:
Mass Spectrometry analysis (RIME, Table S4.1-S4,6)
