# Supplementary material for: Orthogonal proteogenomic analysis identifies the druggable PA2G4-MYC axis in 3q26 AML
Source: Nat Commun. 2024 Jun 4;15:4739. doi: 10.1038/s41467-024-48953-3 (PMC11150407; doi:10.1038/s41467-024-48953-3)
Supplement: Supplementary file 8 — Reporting Summary [file 41467_2024_48953_MOESM8_ESM.pdf]

nature portfolio

Corresponding author(s): Giovanni Roti

Last updated by author(s): Apr 19, 2024

Reporting Summary

Nature Portfolio wishes to improve the reproducibility of the work that we publish. This form provides structure for consistency and transparency in reporting. For further information on Nature Portfolio policies, see our Editorial Policies and the Editorial Policy Checklist.

Statistics

For all statistical analyses, confirm that the following items are present in the figure legend, table legend, main text, or Methods section.

n/a	Confirmed	
☐	☒	The exact sample size (*n*) for each experimental group/condition, given as a discrete number and unit of measurement
☐	☒	A statement on whether measurements were taken from distinct samples or whether the same sample was measured repeatedly
☐	☒	The statistical test(s) used AND whether they are one- or two-sided<br>*Only common tests should be described solely by name; describe more complex techniques in the Methods section.*
☐	☒	A description of all covariates tested
☐	☒	A description of any assumptions or corrections, such as tests of normality and adjustment for multiple comparisons
☐	☒	A full description of the statistical parameters including central tendency (e.g. means) or other basic estimates (e.g. regression coefficient) AND variation (e.g. standard deviation) or associated estimates of uncertainty (e.g. confidence intervals)
☐	☒	For null hypothesis testing, the test statistic (e.g. *F*, *t*, *r*) with confidence intervals, effect sizes, degrees of freedom and *P* value noted<br>*Give P values as exact values whenever suitable.*
☒	☐	For Bayesian analysis, information on the choice of priors and Markov chain Monte Carlo settings
☒	☐	For hierarchical and complex designs, identification of the appropriate level for tests and full reporting of outcomes
☒	☐	Estimates of effect sizes (e.g. Cohen's *d*, Pearson's *r*), indicating how they were calculated

Our web collection on statistics for biologists contains articles on many of the points above.

Software and code

Policy information about availability of computer code

Data collection	Small molecule screening assay:<br>Data was collected with PerkinElmer 2030 Workstation Software v4.0<br><br>Apoptosis and DNA content:<br>Data was collected with Attune Cytometric Software v5.3.2415.0<br><br>Immunohistochemistry:<br>Images data was collected with MoticEasyScan One Software<br><br>Immunodetection:<br>Immunodetection signals were acquired using the LI-COR Odyssey imaging system software
Data analysis	High-throughput in silico screening:<br>Marker genes for the EVI1 "on" vs. "off" signature were chosen using publicly available Affymetrix microarray expression profiling data on TF1 cells transduced with shRNAs targeting EVI1 (E-GEOD 16238) or from mRNA quantification in AML cell lines harboring 3q26 aberration (EMTAB-2225)(Groschel S, et al., Cell 157, 369-381, 2014). For the TF1 study, we then inferred marker genes to the ConnectivityMap (CMap) database (https://clue.io/lincs)(Subramanian A, et al., Cell 171, 1437-1452.e1417, 2017), while for the cross-validation set in EVI1 repressed

HNT34 or AMLHigh vs. AMLLow with the The Library of Integrated Network-Based Cellular Signatures (LINCS)(Pilarczyk M, et al. Nat Commun 13, 4678, 2022). We used the parametric bootstrap method on sets of molecules to calculate the enrichment of compound classes.

Small molecule screening assay:
The effect on cellular viability was defined as normalized percentage of cell death (POC) on the basis of the average of duplicates (POC_molecules) using the following formula: [negative controls luminescence (DMSO) — sample luminescence]/[negative controls luminescence (DMSO)] x 100. Pairwise distances between the given fingerprints of the top candidates and fit a beta distribution to the resulting Tanimoto scores, conditioned on the number of set bits in each fingerprint (ChemmineR package).

Apoptosis and DNA content assays:
Data was processed by FlowJo V10 (Tree Star, LLC, Ashland, OR, USA) analytical software.

Immunofluorescence:
Immunofluorescence images were processed with ImageJ (http://rsbweb.nih.gov/ij/) and Leica Image Compass.

Immunohistochemistry:
Images were quantified using QuPath software v.0.3.2 (https://qupath.github.io/).

ChIP sequencing (ChIPSeq):
For processing sequencing data, samples were analyzed using the of-core ChIP pipeline (version 2.0.0)(Ewels PA, et al., Nat Biotechnol 38, 276-278, 2020) that utilizes MACS2 for peak calling (Zhang Y, et al., Genome Biol 9, R137, 2008) and HOMER for annotation of peaks (Heinz S, et al. Mol Cell 38, 576-589, 2010). Normalized BigWig files were scaled to 1 million mapped reads to be able to compare coverage across multiple samples. Tracks illustrating read coverage and representative peaks were visualized using the IGV genome browser with Human (GRCh37/hg19) genome (Robinson JT, Nat Biotechnol 29, 24-26 2011).

Mass spectrometry:
Tandem mass spectra were extracted and analyzed by PEAKS Studio version 8 build 20. For a label-free quantitation (LFQ) analysis the Thermo raw files were analyzed using MaxQuant (MQ) version 2.4.2.0.

RNA sequencing:
The raw sequence files were quality-controlled using FastQC (v 1.3) (http://www.bioinformatics.babraham.ac.uk/projects/fastqc/, accessed in May 2020). Transcripts were aligned using the STAR package (v2.7.1a) and quantified using the "quantMode GeneCounts" function with ENSEMBL annotation and the human genome version GRCh38 as a reference. Read counts generated by STAR were analyzed using the DESeq2 R package.

Single cell RNA sequencing (scRNASeq):
A digital expression matrix was generated for each sample using Cellranger software (10x Genomics), and the data were integrated using Harmony and analyzed using Seurat (R package)(Hao Y, et al., Cell 184, 3573-3587.e3529, 2021). Cell types were assigned using the Seurat label transferring method and a bone marrow—specific reference (included in the SeuratData package). Conserved markers and differentially expressed genes were identified for each cluster/cell type using the Benjamini-Hochberg method to Adj.P for multiple testing. Statistically significant differentially expressed genes for each cluster/cell type were used to perform a gene set enrichment analysis (GSEA) using the ClusterProfiler package and the Hallmark gene-set from MSigDB. Furthermore, the identification of the leukemic cell population was assessed by computing a transcriptional signature score using the UCell R package.

Next-generation sequencing:
Data were analyzed with Sophia DDM® software version 5.10.11.1 (Sophia Genetics SA).

Statistical analysis:
Statistical analyses were performed using GraphPad Prism 8 or R software.

Image Processing:
All images were processed using Affinity Designer v1.10.6.

For manuscripts utilizing custom algorithms or software that are central to the research but not yet described in published literature, software must be made available to editors and reviewers. We strongly encourage code deposition in a community repository (e.g. GitHub). See the Nature Portfolio guidelines for submitting code & software for further information.

Data

Policy information about availability of data

All manuscripts must include a data availability statement. This statement should provide the following information, where applicable:

- Accession codes, unique identifiers, or web links for publicly available datasets
- A description of any restrictions on data availability
- For clinical datasets or third party data, please ensure that the statement adheres to our policy

Publicly available dataset used in this study are available at https://www.ebi.ac.uk/biostudies/arrayexpress, https://www.ncbi.nlm.nih.gov/geo/, or https://www.cbioportal.org/ (E-GEOD 16238 19, GSE14468 31, GSE13458932, E-MTAB-222515).
Data generated in this study have been deposited in ProteomeXchange Consortium PRIDE repository under accession number PXD038686 (RIME mass spectrometry), NCBI's GEO repository under accession number GSE259221 (HNT34 RNA-seq), GSE220170 (MOLM1 and UCSD/AML1 RNA-seq), GSE256129 (HNT34 ChIP-seq), GSE256130 (PR#002 scRNA-seq), GSE256040 (PDLX_PR#003 scRNA-seq), GSE256076 (PDLX_PR#008 scRNA-seq). The raw FASTQ files from NGS DNA

sequencing (Sophia “Myeloid solution” panel) are available under restriceted access, due to Institutianal policies and privacy laws for sensitive, genomic, and personal data, in the European Genome Archive (EGA) repository at EGAD50000000506. Access for non-commercial academic use can be granted upon an email request to the lead contact (giovanni.roti@unipr.it) within two weeks and is contingent upon a Data Access Agreement between institutions. Data will be available for six month once access has been granted. The remaining data are available within the Article, Supplementary Information or Source Data File. Source data are provided with this paper.

Research involving human participants, their data, or biological material

Policy information about studies with human participants or human data. See also policy information about sex, gender (identity/presentation), and sexual orientation and race, ethnicity and racism.

Reporting on sex and gender	Sex and gender were not considered for the study design.
Reporting on race, ethnicity, or other socially relevant groupings	No race, ethnicity, or other socially relevant groupings were considered for the study design.
Population characteristics	Genotypic profiles, diagnoses and treatment were considered in our experimental settings as described in every test. Population characteristics are described in detail in Supplemental Table S2.
Recruitment	Samples were collected from the Hematology and BMT Unit, Azienda Ospedaliero-Universitaria di Parma, Italy and from the Institute of Hematology and Center for Hemato-Oncology Research, University of Perugia and Santa Maria Della Misericordia Hospital, Perugia, Italy.<br>Samples were collected based on diagnosis of acute myeloid leukemia and genotypic profile: 3q26 status.
Ethics oversight	Ethical Commitee of Department of Medicine and Surgery at Parma University Hospital (protocol number 18249/18/05/2017)

Note that full information on the approval of the study protocol must also be provided in the manuscript.

Field-specific reporting

Please select the one below that is the best fit for your research. If you are not sure, read the appropriate sections before making your selection.

[x] Life sciences [ ] Behavioural & social sciences [ ] Ecological, evolutionary & environmental sciences

For a reference copy of the document with all sections, see nature.com/documents/nr-reporting-summary-flat.pdf

Life sciences study design

All studies must disclose on these points even when the disclosure is negative.

Sample size	For in vivo experiments, we calculated a number of at least 7 mice per group to detect statistically significant difference between groups of 80% a power considering a large effect size. However, due to unforeseen death of mice during the experiments we decided to consider the experiment valid based on the consistency with other similar experiments or based on the clear and statistically significant results.
Data exclusions	No data were excluded from the analysis.
Replication	Data were generated in duplicate or triplicate replicates and repeated three times with similar results.
Randomization	Randomization for in vivo experiments was carried out based on tumor burden detected as circulating human blasts (hCD45+ cells) by cytofluorimetric analysis performed at a minimum of 15 days after transplantation.
Blinding	Experimental data analysis was performed blindly for in vivo experiments. Blinding was not applied in our randomization process.

Reporting for specific materials, systems and methods

We require information from authors about some types of materials, experimental systems and methods used in many studies. Here, indicate whether each material, system or method listed is relevant to your study. If you are not sure if a list item applies to your research, read the appropriate section before selecting a response.

Materials & experimental systems

n/a	Involved in the study
☐	☒ Antibodies
☐	☒ Eukaryotic cell lines
☒	☐ Palaeontology and archaeology
☐	☒ Animals and other organisms
☐	☒ Clinical data
☒	☐ Dual use research of concern
☒	☐ Plants

Methods

n/a	Involved in the study
☐	☒ ChIP-seq
☐	☒ Flow cytometry
☒	☐ MRI-based neuroimaging

Antibodies

Antibodies used

PRIMARY ANTIBODIES:
Cell Signaling Technology, (Danvers MA, USA):
EVI1 (C50E12, #2593)(1:1000 dilution),
β-actin (8H10D10, #3700)(1:1000 dilution),
cleaved caspase 3 (Asp175, #9661)(1:1000 dilution),
MYC (#9402)(1:1000 dilution).

Sigma-Aldrich, (St.Louis, MO, USA):
PA2G4 (#HPA016484, #SAB1402863)(1:1000 dilution).

Abcam (Cambridge, United Kingdom):
histone H3 (acetyl K27) (#ab4729) (1:1000 dilution),
PA2G4 (#ab180602)(1:1000 dilution).

Proteintech (Rosemont, IL, USA):
PA2G4(#66055-1-Ig, #15348-1-Ap)(1:1000 dilution).

Santa Cruz Biotechnology (Dallas, TX, USA):
acetylated -Tubulin (#sc-23950)(1:1000 dilution),
HSP90 (#sc-69703)(1:1000 dilution).

Agilent (Santa Clara, CA, USA):
Ki67 (#IR626)(ready-to-use dilution)

BD Bioscience (Franklyn Lakes, NJ, USA)
hCD45 (#AB11153499, #555482 and #555483), all diluted 1:40)

Cell Marque (Rocklin, CA, USA)
MYC (#395R-18)((ready-to-use dilution)

SECONDARY ANTIBODIES:
LI-COR Biotechnology (Lincoln, NE, USA):
IRDye 680LT goat anti-mouse IgG (#925-68020)(1:10000 dilution),
IRDye 800CW goat anti-rabbit IgG (#925-32211)(1:10000 dilution),
IRDye 680RD goat anti-rabbit IgG (#925-68071)(1:10000 dilution).

Invitrogen (Carlsbad, CA, USA):
Goat anti-Mouse Alexa Fluor™ 488(#A11029)(1:400 dilution),
Goat anti-Rabbit Alexa Fluor™ 568(#A11036)(1:400 dilution).

Invitrogen (Carlsbad, CA, USA):
Goat anti-Rabbit IgG (H+L) Highly Cross-Adsorbed Secondary Antibody, Alexa Fluor™ 568 (#A-11036)(1:400 dilution),
Goat anti-Mouse IgG (H+L) Highly Cross-Adsorbed Secondary Antibody, Alexa Fluor™ 488 (#A-11029)(1:400 dilution).

Validation

All primary antibodies used in this study have been validated by the manufacturer, all information is available on the product website:
EVI1 (C50E12, #2593) https://www.cellsignal.com/products/primary-antibodies/evi-1-c50e12-rabbit-mab/2593
β-actin (8H10D10, #3700) https://www.cellsignal.com/products/primary-antibodies/b-actin-8h10d10-mouse-mab/3700
cleaved caspase 3 (Asp175, #9661) https://www.cellsignal.com/products/primary-antibodies/cleaved-caspase-3-asp175-antibody/9661
MYC (#9402) https://www.cellsignal.com/products/primary-antibodies/c-myc-antibody/9402
PA2G4 (#HPA016484) https://www.sigmaaldrich.com/IT/it/product/sigma/hpa016484
PA2G4 (#SAB1402863) https://www.sigmaaldrich.com/IT/it/product/sigma/sab1402863

histone H3 (acetyl K27) (#ab4729) https://www.abcam.com/en-it/products/primary-antibodies/histone-h3-acetyl-k27-antibody-chip-grade-ab4729
PA2G4 (#ab180602) https://www.abcam.com/en-it/products/primary-antibodies/ebp1-antibody-epr14569b-ab180602
PA2G4(#66055-1-Ig) https://www.ptglab.com/products/PA2G4-Antibody-66055-1-Ig.htm
PA2G4(#15348-1-Ap) https://www.ptglab.com/products/PA2G4-Antibody-15348-1-AP.htm
acetylated -Tubulin (#sc-23950) https://www.scbt.com/it/p/acetylated-alpha-tubulin-antibody-6-11b-1
HSP90 (#sc-69703) https://www.scbt.com/it/p/hsp-90-antibody-4f10
Ki67 (#IR626) https://www.citeab.com/antibodies/3383239-ir626-ki-67-antigen-autostainer-link-48

Eukaryotic cell lines

Policy information about cell lines and Sex and Gender in Research

Cell line source(s)	The human cell lines MOLM1 (#ACC 720), UCSD/AML1 (#ACC 691), HNT34 (#ACC 600), TF1 (#ACC 334), MUTZ-3 (#ACC 295), 293T (#ACC 635), OCI/AML3 (#ACC 582), MOLM13 (#ACC 554), NOMO1 (#ACC 542), OCI/AML2 (#ACC 99), GDM1 (#ACC 87), HL-60 (#ACC 3), SKM1 (#ACC 547) and 5637 (#ACC 35) were purchased from the Leibniz-Institut DSMZ-German collection of microorganisms and cell cultures (Germany). IMS-M2 were previously reported in Chi HT, et al. Leuk Res 34, 261-262 (2010). U937T and U937T_E10 were a kind gift from the Rotraud Wieser laboratory (University of Vienna, Clinic of Medicine I, Waehringer Guertel 18-20, 1090 Vienna, Austria).
Authentication	Cell lines were authenticated by repeated STR Profiling.
Mycoplasma contamination	Cell lines were routinely tested negative for mycoplasma contamination.
Commonly misidentified lines (See ICLAC register)	We have not used any cell lines present in the list of known misidentified cell lines maintained by the International Cell Line Authentication Committee.

Animals and other research organisms

Policy information about studies involving animals; ARRIVE guidelines recommended for reporting animal research, and Sex and Gender in Research

Laboratory animals	6-week-old male and non-obese diabetic, severe combined immune-deficient, interleukin (IL)-2 receptor gamma-deficient mice (NODSCID IL2Rgammanull, NSG).<br>12- to 16-week-old male and female NSG-SGM3 (NSGS) expressing human IL3, GM-CSF (CSF2), and SCF (KITLG).
Wild animals	None.
Reporting on sex	Gender-based analysis was not performed because AML affects both males and females. Male mice are prioritized due to a higher incidence of AML in males compared to females.
Field-collected samples	No field collected samples were used in thus study.
Ethics oversight	All procedures were approved under the MD Anderson (Houston, TX) Institutional Animal Care and Use Committee protocol or the N.682/2019-PR protocol at the University of Parma.

Note that full information on the approval of the study protocol must also be provided in the manuscript.

Clinical data

Policy information about clinical studies
All manuscripts should comply with the ICMJEguidelines for publication of clinical research and a completedCONSORT checklist must be included with all submissions.

Clinical trial registration	No formal clinical trial was performed as the patients were treated under a compassionate use program.
Study protocol	N/A
Data collection	Clinical data was recorded in a Case Report Form.
Outcomes	Standard clinical outcomes were applied (e.g.: Hematological response, survival compared to prior therapies).

Plants

Seed stocks	N/A
Novel plant genotypes	N/A
Authentication	N/A

ChIP-seq

Data deposition

☒ Confirm that both raw and final processed data have been deposited in a public database such as GEO.

☒ Confirm that you have deposited or provided access to graph files (e.g. BED files) for the called peaks.

Data access links
May remain private before publication.

GSE256129 [https://www.ncbi.nlm.nih.gov/geo/query/acc.cgi?&acc=GSE256129]

Files in database submission

GSM8086172 AR2_27AC1
GSM8086173 AR2_27AC2
GSM8086174 AR2_EVI1
GSM8086175 AR2_EVI2
GSM8086176 AR2_Input1
GSM8086177 AR2_Input2
GSM8086178 DMSO_27AC1
GSM8086179 DMSO_27AC2
GSM8086180 DMSO_EVI1
GSM8086181 DMSO_EVI2
GSM8086182 DMSO_Input1
GSM8086183 DMSO_Input2
GSM8086184 ENT_27AC1
GSM8086185 ENT_27AC2
GSM8086186 ENT_EVI1
GSM8086187 ENT_EVI2
GSM8086188 ENT_Input1
GSM8086189 ENT_Input2

Raw fastq and processed bigWig files are provided for each sample.

Genome browser session
(e.g. UCSC)

No longer applicable.

Methodology

Replicates	n=2
Sequencing depth	50 million reads/sample
Antibodies	EVI1 (Cell Signaling Technology, #2593S)<br>H3K27Ac (Abcam, #ab4729)
Peak calling parameters	--narrow_peak --macs_fdr 0.05
Data quality	MACS2 was used for peak calling. Number of peaks at 5% FDR depends on the sample type, and are comprised between 57000 and 118000
Software	For processing sequencing data, samples were analyzed using the of-core ChIP pipeline (version 2.0.0)(Ewels PA, et al., Nat Biotechnol 38, 276-278, 2020) that utilizes MACS2 for peak calling (Zhang Y, et al., Genome Biol 9, R137, 2008) and HOMER for annotation of peaks (Heinz S, et al. Mol Cell 38, 576-589, 2010). Normalized BigWig files were scaled to 1 million mapped reads to be able to compare coverage across multiple samples. Tracks illustrating read coverage and representative peaks were visualized using the IGV genome browser with Human (GRCh37/hg19) genome (Robinson JT, Nat Biotechnol 29, 24-26 2011).

Flow Cytometry

Plots

Confirm that:

- [x] The axis labels state the marker and fluorochrome used (e.g. CD4-FITC).
- [ ] The axis scales are clearly visible. Include numbers along axes only for bottom left plot of group (a 'group' is an analysis of identical markers).
- [x] All plots are contour plots with outliers or pseudocolor plots.
- [x] A numerical value for number of cells or percentage (with statistics) is provided.

Methodology

Sample preparation	Standard protocol for apoptosis as described in the manuscript.
Instrument	Thermo Fisher Scientific Attune NxT.
Software	Attune Cytometric Software v5.3.2415.0, FlowJo software v10.
Cell population abundance	n≥20000
Gating strategy	Gating strategy based on positive and negative controls.

- [ ] Tick this box to confirm that a figure exemplifying the gating strategy is provided in the Supplementary Information.
